# Cross-modal predictive modeling of multi-omic data in 3D airway organ tissue equivalents during viral infection

**DOI:** 10.3389/fgene.2025.1658577

**Published:** 2025-09-25

**Authors:** Mostafa Rezapour, Patrick M. McNutt, David A. Ornelles, Stephen J. Walker, Sean V. Murphy, Anthony Atala, Metin Nafi Gurcan

**Affiliations:** ^1^ Center for Artificial Intelligence Research, Wake Forest University School of Medicine, Winston-Salem, NC, United States; ^2^ Wake Forest Institute for Regenerative Medicine, Wake Forest University School of Medicine, Winston-Salem, NC, United States; ^3^ Microbiology Immunology, Wake Forest University School of Medicine, Winston-Salem, NC, United States

**Keywords:** predictive modeling, 3D airway organ tissue equivalent (OTEs), viral infection, RNA-Seq data, NanoString technologies, differential expression analysis

## Abstract

**Introduction:**

Developing robust predictive models from multi-omics data is challenging because sample sizes are typically small (often fewer than 100) while the feature space is vast (over 20,000 molecular features such as genes, transcripts, and proteins), which increases the risk of overfitting and limits generalizability. To address this challenge, this study introduces the Magnitude-Altitude Score Analysis for Tracking Infection and Time-Dependent Genes (MASIT), a novel method adept at filtering out irrelevant features/genes while focusing on important ones.

**Methods:**

Applied to the 3D airway organ tissue equivalent model that mimics human airway physiology, MASIT employed both RNA-Seq and NanoString technologies for a comprehensive analysis. RNA-Seq offered a transcriptomic overview of 19,671 protein coding genes, whereas NanoString targeted 773 specific genes. We used MASIT to analyze gene expression changes in the airway tissue equivalent after exposure to Influenza A virus, Human metapneumovirus, and Parainfluenza virus type 3 at 24- and 72-hour post-infection. MASIT was trained and validated on NanoString data, tested on the held-out RNA-Seq test set, and benchmarked against widely used feature selection approaches, including Fisher score, minimum Redundancy Maximum Relevance, embedded Lasso regression, and Boruta feature importance.

**Results:**

MASIT achieved a 92% accuracy in differentiating eight groups of infected samples. Our findings showed that MASIT outperformed models using the full gene set, notably in algorithms like Random Forest, XGBoost, and AdaBoost. Selected genes such as IFIT1, IFIT2, IFIT3, OASL, IFI44, and OAS3 were particularly effective in categorizing samples by viral type and infection stage. Benchmarking further demonstrated that MASIT not only exceeded the performance of existing feature selection methods within NanoString data but also uniquely maintained high accuracy and stability when applied to held-out RNA-Seq data.

**Discussion:**

These results provide insights into the host’s molecular response to viral infections and highlight MASIT as a robust tool for analyzing high-dimensional, small-sample multi-omics datasets.

## 1 Introduction

The human bronchial tree is important not only for air conduction but also for its role as a key interface for several biological functions. It enables mucociliary clearance, airway humidification, and the detection and defense against pathogens and particulates, significantly impacting the immune system (Crystal et al., 2008). The diversity in airway epithelium, which includes cell types such as ciliated cells, goblet cells, and basal cells (Knight and Holgate, 2003), is directly linked to respiratory diseases such as asthma and chronic obstructive pulmonary disease (COPD) (Hiemstra et al., 2015). This epithelial functionality is augmented by its interactions with the surrounding microenvironment, including various cell types and the extracellular matrix (ECM), which are important for maintaining structural integrity and cellular communication within the airways (Tam et al., 2011; Sacco et al., 2004; Liu et al., 2021).

Traditional models using monocultures of human bronchial epithelial (HBE) cells on porous polymer membranes at air-liquid interface (ALI) have been beneficial for studying certain airway functions like mucus production and ciliation. However, these models lack comprehensive cell-cell interactions with non-epithelial cells and do not adequately mimic the complex cell-ECM interactions. Additionally, the typically stiffer growth surfaces can alter the phenotype, diversity, and functionality of HBE cells, thus not accurately reflecting the conditions of live airway epithelia (Hirst et al., 2014; Jaroch et al., 2018; Barros et al., 2021).

Our group has developed an innovative planar airway 3D organ tissue equivalent (OTE) model that significantly enhances the simulation of human airway physiology *in vitro* (Leach et al., 2023). This model integrates a well-differentiated HBE layer maintained at an ALI on a hydrogel substrate, which supports the inclusion of native lung fibroblasts and solubilized human lung extracellular matrix (ECM) (Leach et al., 2023). This configuration allows for a more realistic representation of the physiological interactions between cells and the ECM, as well as biomechanical influences that are important for studying airway function and disease (Leach et al., 2023).

Our OTE model addresses the traditional models’ limitations by providing a dynamic 3D microenvironment where cellular interactions and ECM mechanics more closely resemble those found *in vivo*. This advanced setup not only supports the differentiation and functional activities of airway epithelial cells but also facilitates intricate studies on how these cells interact with other airway components under physiological and pathological conditions (Leach et al., 2023).

In our recent study, we investigated the gene expression dynamics within 3D airway OTEs following infections by Influenza A virus (IAV), Human metapneumovirus (MPV), and Parainfluenza virus type 3 (PIV3) at both 24- and 72-h post-infection. Using Generalized Linear Models (GLMs) (Nelder and Wedderburn, 1972) with Quasi-Likelihood F-tests (QL) (Wedderburn, 1974) alongside the novel Magnitude-Altitude Score (MAS) and Relaxed Magnitude-Altitude Score (RMAS) algorithms, we analyzed RNA-Seq data across 19,671 genes (Rezapour et al., 2024a). This approach enabled the identification of genes significantly altered by viral infection, with an emphasis on those important for initiating and sustaining the host’s immune response. The Gene Ontology (GO) analysis provided a detailed view of how IAV, MPV, and PIV3 impact vital biological processes and cellular components, and highlighted a strategic adaptation of cellular functions to support viral replication. Notably, the activation of interferon-stimulated genes such as *IFIT1*, *IFIT2*, *IFIT3*, and *OAS1*, along with alterations in cilium and mitochondrial ribosome assembly, underscored a robust antiviral response and the manipulation of host machinery to favor viral survival (Rezapour et al., 2024a).

In another study, we used the 3D airway OTE model but employed the NanoString platform instead of RNA-Seq for gene expression analysis, focusing on 773 specific genes (Rezapour et al., 2024b). This shift to NanoString technology was driven by its capability to provide precise and direct quantification of mRNA transcripts, which is important for accurately capturing the nuances of gene expression changes at predefined time points post-infection. We employed the Magnitude-Altitude Score (MAS) algorithm, an analytical tool designed to integrate biological significance with statistical rigor. The MAS algorithm not only identified genes with substantial fold changes but also applied the Benjamini-Hochberg correction to control for false discoveries, enhancing the reliability of our data (Rezapour et al., 2024b). This dual approach of using NanoString, coupled with the MAS algorithm, allowed us to unveil distinct patterns of gene expression in response to viral infections. At 24-h post-infection, a pronounced interferon-stimulated gene response was evident, particularly against IAV, showcasing an immediate robust antiviral defense. Meanwhile, MPV and PIV3 infections illustrated different aspects of the immune response, emphasizing the versatility of the host’s defensive strategies. By 72 h, the evolving gene expression profiles indicated an adaptation to ongoing viral presence, with shifts towards maintaining a balanced state of antiviral defense and cellular homeostasis.

We also conducted a comparative analysis of RNA-Seq and NanoString platforms to evaluate their efficacy in profiling gene expression within the same 3D airway OTE model under various viral infections at 24- and 72-h post-infection (Rezapour M. et al., 2024). The comprehensive evaluation employed a range of analytical techniques including Spearman correlation, Distance correlation, and Bland-Altman analysis, along with the use of Generalized Linear Models (GLMs), Huber regression, and the concordance analysis. Our findings highlighted a high degree of agreement between the platforms, particularly in their capacity to identify crucial antiviral defense genes such as *ISG15*, *MX1*, *RSAD2*, and the *OAS* family.

Building on our extensive research using our innovative 3D OTE model (Leach et al., 2023), this paper aims to further elucidate gene expression dynamics across different viral infections and post-infection time points using RNA-Seq and NanoString technologies. Unlike our previous studies (Rezapour et al., 2024a; Rezapour et al., 2024b; Rezapour M. et al., 2024), which employed these platforms for differential expression analysis and biomarker identification across entire datasets, the primary objective of this paper is to develop a novel predictive model tailored for multi-omics data with limited sample sizes, such as six samples per group.

The challenge with employing complex machine learning models in scenarios with small sample sizes is their tendency to overfit (Dietterich, 1995). Overfitting occurs because these models have a large number of parameters relative to the amount of available training data, which leads them to learn noise and anomalies in the data as if they were meaningful patterns. This not only prevents the model from generalizing to new datasets but often results in poor training performance as the model fails to converge on a stable solution. To address these issues, we introduce a novel filtering system that precedes the application of different classifiers such as Logistic Regression (LR) (Kleinbaum et al., 2002; Christodoulou et al., 2019), Support Vector Machine (SVM) (Pisner and Schnyer, 2020), Naive Bayes (Yang, 2018), Random Forest (RF) (Speiser et al., 2019), XGBoost (Chen and Guestrin, 2016), AdaBoost (Hastie et al., 2009), Gradient Boosting Machine (GB) (Friedman, 2001), Extremely Randomized Trees (ER) (Geurts et al., 2006) and k-Nearest Neighbors (kNN) (Guo et al., 2003). This system, namely Magnitude-Altitude Score Analysis for Tracking Infection and Time-Dependent Genes (MASIT) is designed to carefully select transcripts that are most indicative of different viral infection conditions and their respective post-infection times.

The MASIT strategically reduces the number of input features to focus on genes identified through rigorous statistical testing, which addresses the challenge of training models on comprehensive genomic datasets with limited sample sizes. By prioritizing statistically robust features, we reduce the risk of overfitting and enhance the reliability and interpretability of our predictive models. Here, we outline the key contributions of our study:

• Our study introduces the first supervised predictive model that leverages stringent differential expression analysis for gene selection. This model uniquely combines predictive functionality with statistical rigor and focuses on genes whose expression patterns are strongly associated with viral infection responses and treatment effects. By ensuring that the selected genes demonstrate both substantial effect sizes (fold changes) and statistical significance, we enhance the interpretability and applicability of our predictive outcomes.
• Addressing a common challenge in the field, our work pioneers a predictive modeling approach tailored for multi-omics datasets characterized by small sample sizes. Through the development of a novel pre-filtering system that precedes a classifier, our model effectively minimizes overfitting. This is important in scenarios where the number of parameters could easily overwhelm the amount of available data, thus preserving the integrity and reliability of the model’s predictions. Our approach sets a new standard for handling limited-sample datasets in biomedical research, offering a robust framework that can be adapted to various diseases and conditions.
• Uniquely, our novel filtering system, MASIT, is trained and validated on NanoString data and tested using a held-out RNA-Seq dataset, marking a first in the field. This cross-modal validation not only demonstrates the filtering system’s adaptability and robustness across different technological platforms but also underscores its accuracy and effectiveness in diverse experimental settings.

## 2 Materials and methods

In this study, we carefully prepared the virus infection medium and conducted a series of controlled infections on our 3D airway OTE model (Leach et al., 2023) with three different viruses: IAV, MPV, and PIV3. The infection process was standardized by involving the preparation of the infection medium, sterilization, and specific conditions for incubation and viral application to the OTEs. Following the infection, RNA was extracted using a protocol that ensured the removal of potential DNA contaminants, where it preserved the integrity of our samples for detailed molecular analysis. The extracted RNA underwent sequencing and analysis. We used RNA sequencing to construct cDNA libraries, which were then processed and sequenced on an Illumina^®^ NovaSeq 6000 System. Additionally, we applied the NanoString nCounter^®^ Analysis System for highly multiplexed detection of mRNA targets, which is especially suitable for samples where RNA integrity may vary. For data normalization and analysis, we used the Trimmed Mean of M-values (TMM) method for RNA-Seq and a two-step process involving Positive Control and CodeSet Content Normalization for NanoString data. These procedures, important for accurately dissecting the complex interactions within the OTE model, are described in full detail in (Rezapour M. et al., 2024).

Table 1 illustrates the eight groups of infected OTEs classified based on (1) virus and (2) post-infection time within both NanoString and RNA-Seq data. Our primary goal is to identify gene biomarkers using the NanoString data and subsequently validate these biomarkers within the held-out RNA-Seq data. This method allows us to directly assess whether the identified biomarkers from NanoString hold up under the broader transcriptomic scrutiny provided by RNA-Seq, without the intermediary step of training. The rationale for this approach stems from the technological complementarity of NanoString and RNA-Seq.

**TABLE 1:** Eight infection conditions were classified based on (1) virus and (2) post-infection time within both NanoString and RNA-Seq data.

Primary condition		# samples	Virus	Post-infection time	Primary condition		# samples	Virus	Post-infection time
1	Mock-24	6	Mock	24-h	5	Mock-72	6	Mock	72-h
2	IAV-24	6	IAV	24-h	6	IAV-72	6	IAV	72-h
3	MPV-24	6	MPV	24-h	7	MPV-72	6	MPV	72-h
4	PIV3-24	6	PIV3	24-h	8	PIV3-72	6	PIV3	72-h

### 2.1 Magnitude-altitude score (MAS)

In the field of differential gene expression analysis, accurately identifying genes that signal specific conditions is very important. Traditional methods often focus solely on statistical significance (measured by adjusted p-values) or only on effect size (measured by fold change), potentially overlooking the importance of integrating both aspects. This may lead to the selection of genes that, despite showing large expression changes, lack statistical support, or *vice versa*. To address this limitation, we employed the Magnitude-Altitude Score (MAS) algorithm [see Algorithm 1 in (Rezapour et al., 2024b)] in our previous studies (Rezapour et al., 2024a; Rezapour et al., 2024b; Rezapour M. et al., 2024; Rezapour et al., 2024d). The MAS algorithm integrates effect size (captured by fold change) with statistical significance (adjusted p-values), which ensures that the identified genes are both statistically robust and strongly altered in expression. This dual consideration offers a more balanced and comprehensive perspective on gene expression. Notably, the MAS algorithm has already demonstrated superior performance in complex problems, such as identifying axillary lymph node metastasis in breast cancer (Rezapour et al., 2024d), outperforming existing state-of-the-art methods.

Initially, for data such as from NanoString, where assumptions of normality and equal variance are reasonable, MAS performs a two-sample t-test (α = 0.05) (Kim, 2015) between Baseline (mock-infected samples) and Treated (infected) conditions for each gene to detect differences in expression. The p-values from these tests are then adjusted using the Benjamini-Hochberg method (Benjamini et al., 1995) to control the false discovery rate. For genes that show significant differential expression, the algorithm calculates fold changes, which provides a measure of the magnitude of expression change associated with the treatment. The MAS assigns a score to significant genes using the formula:

$${\text{MAS}}_{l}=\left|{(\log_{2}(\text{FC}}_{l}){|}^{M}\right|{\left(\log_{10}(p\right.}_{l}^{\text{BH}}))\;{|}^{A},$$

where $p_{l}^{BH}$ denotes BH adjusted p-values for l =1,2,…,s , where s is the number of rejected null hypotheses based on BH adjusted p-value ( $\left.p_{l}^{BH}<\alpha =0.05\right)$ . The hyperparameters M and A, both set to 1 in this study, are chosen to balance the p-value and the log fold change. It then assigns a rank to each significant gene such that the gene with the maximum RMAS score is ranked 1. The final ranking of genes based on their MAS scores highlights those with the most substantial evidence of a treatment effect. The output lists these BH-significant genes along with their MAS ranks and scores, providing a prioritized view of the treatment’s impact.

The MAS algorithm can be concisely represented as a function/mapping, $\text{MAS }\left(B,T,M,A\right)=\left(G,R,\Upsilon \right),$ where B and T denote Baseline and Treated gene expressions, respectively, and M and A are hyper-parameters, G is the list of BH-significant genes, R provides their MAS ranks and Υ contains their MAS scores. In the MAS algorithm, the hyper-parameters M and A play key roles in the computation of the MAS score.

### 2.2 Magnitude-altitude score analysis for tracking infection and time-dependent genes (MASIT)

In this section, we introduce the Magnitude-Altitude Score Analysis for Tracking Infection and Time-Dependent Genes (MASIT) algorithm (Algorithm 1), designed to enhance our understanding of how gene expression evolves in response to infection over time. Unlike our previous studies (Rezapour et al., 2024a; Rezapour et al., 2024b; Rezapour M. et al., 2024) that primarily focused on identifying highly expressed genes across various conditions, MASIT is engineered to detect and analyze genes that can distinctly differentiate between multiple infection states and their corresponding post-infection timelines.

The primary objective of MASIT is to identify BH-significant genes capable of distinguishing various active infection conditions and their respective post-infection times. This involves assessing gene expressions from a baseline (mock) and multiple treated (infected) groups, each treated with a different virus, at specific post-infection time points. The unique aspect of this algorithm is its ability to not only differentiate each treated group from the baseline at each post-infection time but also to distinguish between each pair of treated groups across distinct time points.

For instance, in a scenario where we have multiple treated groups of OTEs, each actively infected with a distinct virus, and a baseline group (mock), the algorithm assesses the expression data at various post-infection times $T_{1},T_{2},...,T_{t}$ . Our initial and primary aim is to identify at least one gene, infection-dependent, within each post-infection time $T_{i}$ that can differentiate all treated groups from each other and from the baseline at that specific time $T_{i}$ . Subsequently, for predictive purposes, we seek time-dependent genes, which are used solely for tracking post-infection time points. Biologically, the time-dependent genes may or may not be directly related to the infection but are important for determining the post-infection time. It is important to note that the combination of infection-dependent and time-dependent genes provides a comprehensive picture of whether a sample is infected with a specific virus and at which time.

Algorithm 1 Magnitude-Altitude Score Analysis for Tracking Infection and Time-Dependent Genes (MASIT).

**Input**: Gene expressions for
   • ${Baseline}_{T_{i}}$ *:* Control group at time $T_{i}$ , for i=1,2,...,t.

   • ${Treated}_{T_{i}}^{X}$ *:* Group of OTEs infected by the ${ⱱ}^{th}$ virus at time $T_{i}$ , for ⱱ=1,2,...,q, and i=1,2,...,t.

   • Set $G_{Infection}=\left\{\right\}$ , $G_{Time}=\left\{\right\}$ .
**Step 1 :**  Identify commonly BH-Significant genes at each time:
   **For** i=1 to t :
   • **For** ⱱ=1 to q :
      
$\left({G_{T_{i}}^{ⱱ}R}_{T_{i}}^{ⱱ},\Upsilon_{T_{i}}^{ⱱ}\right)$ = MAS ( ${Baseline}_{T_{i}}$ ; ${Treated}_{T_{i}}^{ⱱ}$ , M = 1, A = 1),
    **End (For)**
   • Set $G_{T_{i}}^{MAS\;Common}$ = $\overset{q}{\underset{ⱱ=1}{⋂}}G_{T_{i}}^{ⱱ}$ , and construct a $\tau_{i}^{\prime }\times \left(q+1\right)$ matrix, $\Gamma_{T_{i}}=\left[G_{T_{i}}^{MAS\;Common},R_{T_{i}}^{u_{1}}{,R}_{T_{i}}^{u_{2}}{,...,R}_{T_{i}}^{u_{q}}\right]$ , where $G_{T_{i}}^{MAS\;Common}$ is the set of $\tau_{i}^{\prime }$ common BH-significant genes among all treated groups (infected OTEs) at time $T_{i}$ , and $R_{T_{i}}^{u_{j}}\subseteq R_{T_{i}}^{j}$ is the corresponding MAS ranks vector for the genes in $G_{T_{i}}^{MAS\;Common}$ for viruses j = 1, 2, … , q, at post-infection time $T_{i}$ .
   • Solve $k^{*}=\underset{1\leq k\leq \tau_{i}^{\prime }}{\arg\;\min\;}\left(\max{\left\{R\right.}_{T_{i}}^{u_{1k}},R_{T_{i}}^{u_{2k}},...,R_{T_{i}}^{u_{qk}}\}\;\right)$ , where $R_{T_{i}}^{u_{jk}}$ is the $k^{th}$ entry of $R_{T_{i}}^{u_{j}}.$

   • Select $g_{T_{i}}^{Infection}={\left(G_{T_{i}}^{MAS\;Common}\right)}_{k^{*}}$ , where ${\left(G_{T_{i}}^{MAS\;Common}\right)}_{k^{*}}$ is the $k^{th}$ entry of $G_{T_{i}}^{MAS\;Common}$ .
   • $G_{Infection}=G_{Infection}\cup \left\{g_{T_{i}}^{Infection}\right\}$ .
   **End (For)**
**Step 2 :**  Selecting the time-related genes:
   **For** i=1 to t−1 :
    **For** j=i+1 to t :
     • $\left({G_{T_{i}\to T_{j}}^{Baseline},R}_{T_{i}\to T_{j}}^{Baseline},\Upsilon_{T_{i}\to T_{j}}^{Baseline}\right)$ = MAS ( ${Baseline}_{T_{i}}$ ; ${Baseline}_{T_{j}}$ , M = 1, A = 1).
     • **For** ⱱ=1 to q :
      
$\left({G_{T_{i}\to T_{j}}^{ⱱ}R}_{T_{i}\to T_{j}}^{ⱱ},\Upsilon_{T_{i}\to T_{j}}^{ⱱ}\right)$ = MAS ( ${Treated}_{T_{i}}^{ⱱ},{Treated}_{T_{j}}^{ⱱ}$ , M = 1, A = 1),
      **End (For)**
      • Set $G_{T_{i}\to T_{j}}^{Time}$ = $\left(\overset{q}{\underset{ⱱ=1}{⋂}}G_{T_{i}\to T_{j}}^{ⱱ}\right)\cap G_{T_{i}\to T_{j}}^{Baseline}$ , and construct a $\tau_{i}^{\prime \prime }\times \left(q+2\right)$ matrix, ${ϒ}_{T_{i}\to T_{j}}=\left[G_{T_{i}\to T_{j}}^{Time},R_{T_{i}\to T_{j}}^{u_{1}}{,R}_{T_{i}\to T_{j}}^{u_{2}},...,R_{T_{i}\to T_{j}}^{u_{q}},R_{T_{i}\to T_{j}}^{u_{Baseline}}\right]$ , where $G_{T_{i}\to T_{j}}^{Time}$ is the set of $\tau_{i}^{\prime \prime }$ common BH-significant time-dependent genes among all groups between time $T_{i}$ and $T_{j}$ , and $R_{T_{i}\to T_{j}}^{u_{j}}\subseteq R_{T_{i}\to T_{j}}^{j}$ and $R_{T_{i}\to T_{j}}^{u_{Baseline}}\subseteq R_{T_{i}\to T_{j}}^{Baseline}$ are subsets of the original genes’ ranks corresponding to commonly MAS-selected genes.
     • Solve $k^{*}=\underset{1\leq k\leq \tau_{i}^{\prime \prime }}{\arg\;\min\;}\left(\max{\left\{R\right.}_{T_{i}\to T_{j}}^{u_{1k}},R_{T_{i}\to T_{j}}^{u_{2k}},...,R_{T_{i}\to T_{j}}^{u_{qk}},R_{T_{i}\to T_{j}}^{u_{Baseline}}\}\;\right)$ , where $R_{T_{i}\to T_{j}}^{u_{jk}}$ is the $k^{th}$ entry of $R_{T_{i}\to T_{j}}^{u_{j}}.$

     • Select $g_{T_{i}\to T_{j}}^{Time}={\left(G_{T_{i}\to T_{j}}^{Time}\right)}_{k^{*}}$ , where ${\left(G_{T_{i}\to T_{j}}^{Time}\right)}_{k^{*}}$ is the $k^{th}$ entry of $G_{T_{i}\to T_{j}}^{Time}$ .
     • $G_{Time}=G_{Time}\cup \left\{g_{T_{i}\to T_{j}}^{Time}\right\}$ .
    **End (For)**
   **End (For)**
**Output:** The set of distinctive genes $G_{Selected}=G_{Infection}\cup G_{Time}$ .

The final output of MASIT, a set of distinctive genes categorized under $G_{\text{Selected}}$ provides a robust basis for enhancing predictive models. By focusing on both the specificity of infection-related changes and their temporal dynamics, these features substantially enhance the predictive power of our models. Depending on the number of samples, we can adjust the number of selected genes. We adhere to the rule of thumb that suggests using one feature for every 10 samples to ensure the model does not overfit and remains generalizable (Ying, 2019). In our study, due to the limited number of samples, which is 48 with 6 per class, we only select one gene for each post infection time point and one for temporal classification during the predictive modeling.

MASIT operates under the assumption that the gene sets identified by MAS as common BH-significant among all treated groups and baseline conditions are non-empty. In scenarios where no common BH-significant genes are found, MASIT can adapt by relying on raw p-values to continue its analysis. Given the robust performance of MAS and MASIT, which are designed to identify the top gene demonstrating the highest statistical significance, we can further relax the significance level if raw p-values yielded non-empty sets.

Additionally, the degree of similarity between the conditions of the treated groups is expected to be sufficient to allow the identification of non-empty sets of significant genes under BH-correction, or at the very least, using raw p-values. In our study, the inclusion of three viral infection conditions (IAV, MPV, and PIV3) under NanoString data (see Sections 2.3, 2.4) allowed us to fully apply MASIT using the Benjamini-Hochberg method without encountering any empty sets.

### 2.3 Cross-modal capability of MASIT

To explore the cross-modal capability of MASIT across NanoString and RNA-Seq platforms, we initially apply MASIT to the NanoString data, without evaluating their predictive capabilities. From this analysis, we identify approximately 1% of the genes, specifically 8 key markers. These genes are classified into two categories: *infection-dependent* and *time-dependent* genes.

*Infection-dependent* genes are identified based on their significant expression changes under viral infection conditions. These genes are biologically meaningful as they play key roles in mediating the OTE’s response to viral infections and provide key insights into the biological interpretation of viral infection dynamics. *Time-dependent* genes, on the other hand, are selected for their ability to enhance classification accuracy by marking specific post-infection time points. While Time-dependent genes are important for improving the temporal classification of the samples, they do not necessarily hold significant biological relevance in terms of the infection process itself. Their inclusion is primarily aimed at refining the model’s ability to differentiate between various stages of infection based on gene expression patterns, thereby improving the overall accuracy of the classification system.

We proceed to test the effectiveness of these 8 selected genes in clustering RNA-Seq samples. Hierarchical clustering (Murtagh and Contreras, 2012) was performed using the Ward’s method (Saraçli et al., 2013) with Euclidean distance as the metric. This analysis aims to validate whether these genes can effectively group the samples based on similarities in their expression profiles across platforms, which confirms their discriminative power and relevance in broader genomic studies.

### 2.4 Cross-modal predictive modeling with MASIT

In this section, we employ the MASIT within a cross-modal predictive modeling framework to analyze gene expression dynamics influenced by viral infections at various post-infection times. Our methodology comprises two primary phases: training and validating of MASIT on NanoString data, followed by testing on the held-out RNA-Seq data.

Initially, we categorize the NanoString data according to different post-infection times, each consisting of baseline and various treated conditions. We undertake a training process using stratified K-fold cross-validation (Wong and YehPo-Yang, 2019) to ensure that our model robustly captures the nuances of the data while preventing overfitting. During each fold of the cross-validation, MASIT is applied solely to the training set to identify a selected group of genes. These genes serve as potential biomarkers and predictors to distinguish between different infection states and timings. These identified genes are subsequently employed to train several models such as logistic regression (Kleinbaum et al., 2002) using one-versus-one (ovo) and one-versus-all (ovr) strategies (Rocha and Goldenstein, 2013), Support Vector Machines (SVM) (Steinwart and Christmann, 2008) with a linear kernel (Cristianini and Shawe-Taylor, 2000), Naive Bayes (Yang, 2018), Random Forest (RF) (Speiser et al., 2019), XGBoost (Chen and Guestrin, 2016), AdaBoost (Hastie et al., 2009), Gradient Boosting Machine (GB) (Friedman, 2001), Extremely Randomized Trees (ER) (Geurts et al., 2006) and k-Nearest Neighbors (kNN) (Guo et al., 2003).

To minimize overfitting, for models such as Random Forest, XGBoost, AdaBoost, Extremely Randomized Trees, and Gradient Boosting, we not only limit the number of genes to three MASIT-chosen genes but also use a limited number of trees and a restricted maximum depth. For the AdaBoost model, although it typically uses decision stumps (trees with a maximum depth of 1) by default, we use decision trees with varying maximum depths to enhance the model’s capacity to capture more complex patterns in the data.

After optimizing and validating our model with NanoString data, we used the MASIT-selected genes as input features for classifiers from the training-validation stage to test them on the held-out RNA-Seq data using K-fold cross-validation. The goal of using cross-validation on RNA-Seq data is to fine-tune the classifiers' parameters to accommodate the different scales of NanoString and RNA-Seq data.

**FIGURE 1:**
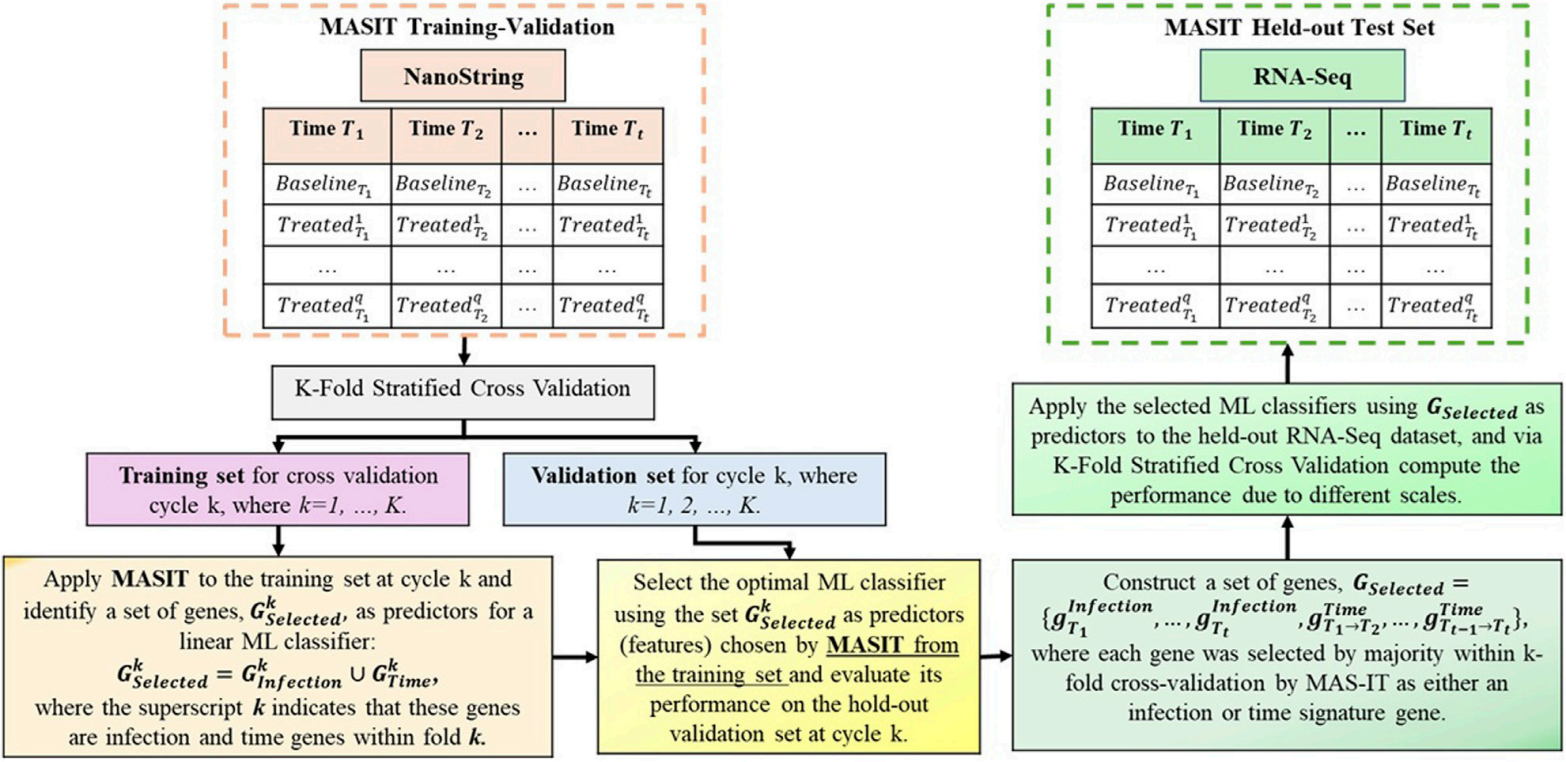
This figure illustrates the two-phase process of training and validating of on NanoString data followed by testing on the held-out RNA-Seq data. It highlights the categorization of data by post-infection times, the application of MASIT for gene selection during training, and the use of optimal linear classifiers for cross-validation and final testing to evaluate the translational efficacy of predictive insights across different genomic technologies.

Note that we could standardize the scales of both NanoString and RNA-Seq data to directly apply the trained NanoString model to RNA-Seq. However, we have chosen to retain the original scales to ensure that the model can accurately identify the virus and post-infection times in new samples from either platform without relying on any transformations. Figure 1 illustrates our structured approach.

For specifying hyperparameters of tree-based models, we employed two complementary strategies. Because of the limited number of samples, we first constrained model capacity by fixing the number of estimators and maximum depth to the lowest feasible values. This conservative approach reduces the risk of overfitting and ensures stability when data availability is highly restricted. However, fixing hyperparameters *a priori* may also prevent models from capturing informative patterns. To address this limitation, we also implemented a nested cross validation procedure. In this framework, the dataset was first split into six outer folds using stratified partitioning to preserve the class distribution. For each outer iteration, five folds were used for training and one was held out for outer testing. Within each outer training set, a second level of five fold inner cross validation was performed, where a grid search was used to evaluate all combinations of the number of estimators and maximum depth. For every parameter combination, inner training accuracy was defined as the mean performance of the model on the training portion of the inner folds, and inner validation accuracy as the mean score on the corresponding validation folds.

The combination of parameters that maximized the inner validation accuracy was then selected as the “inner optimal” configuration. This configuration was subsequently refitted on the entire outer training set and evaluated on the outer test fold to yield the outer validation accuracy, which serves as an unbiased estimate of generalization. The process was repeated across all outer folds, and mean and standard deviation values for training, inner validation, and outer validation accuracies were computed to summarize central tendency and variability.

This design allowed us to systematically evaluate model capacity and identify optimal configurations for Random Forest, Extra Trees, Gradient Boosting, AdaBoost, and XGBoost classifiers. While the fixed minimal-capacity approach is more appropriate in small-sample contexts, nested cross-validation provides a more flexible and rigorous framework for hyperparameter optimization. By combining both approaches, we ensured that our findings were not dependent on arbitrary parameter choices, while maintaining methodological robustness under limited data conditions.

Finally, to evaluate the practical advantages of MASIT, we conducted a systematic benchmarking analysis against widely adopted feature selection strategies. Feature selection methods are generally categorized as filtering, wrapper, embedded, or hybrid approaches (Chandrashekar and Sahin, 2014). In this study, we selected one representative method from each category: Fisher score (filtering) (Sun et al., 2021), minimum Redundancy Maximum Relevance (mRMR, wrapper) (Peng et al., 2005), embedded Lasso regression (Tibshirani, 1996), and Boruta feature importance (hybrid) (Kursa and Rudnicki, 2010). These methods have been extensively applied in gene expression studies and thus provide a relevant basis for comparison. In this comparative framework, we replaced the MAS component of MASIT with each of these methods while keeping the overall structure identical. For all benchmarking experiments, we employed Random Forest as the common downstream classifier. Random Forest was chosen because it is widely used in transcriptomic analyses and handles high-dimensional data effectively. Using a single classifier ensured that observed differences in performance could be attributed to the feature selection strategies themselves rather than classifier-specific effects.

## 3 Results

### 3.1 Cross-modal capability of MASIT

In this section, we aim to apply the MASIT algorithm across the entire NanoString dataset, which includes expression data for 773 genes from OTEs infected with IAV, MPV, and PIV3 at two post-infection times, $T_{1}=24$ hours and $T_{2}=72$ hours. As previously outlined in Section 2.3, our goal is to identify a select group of transcripts, specifically, 8 genes, representing 1% of the total genes within NanoString. These genes will be used to perform hierarchical clustering within the RNA-Seq data. To do so, we identify three top infection genes at time $T_{1}=24$ , and three infection-dependent genes at time $T_{2}=72$ , and two top time-dependent genes for tracking post infection time based on MASIT.

**FIGURE 2:**
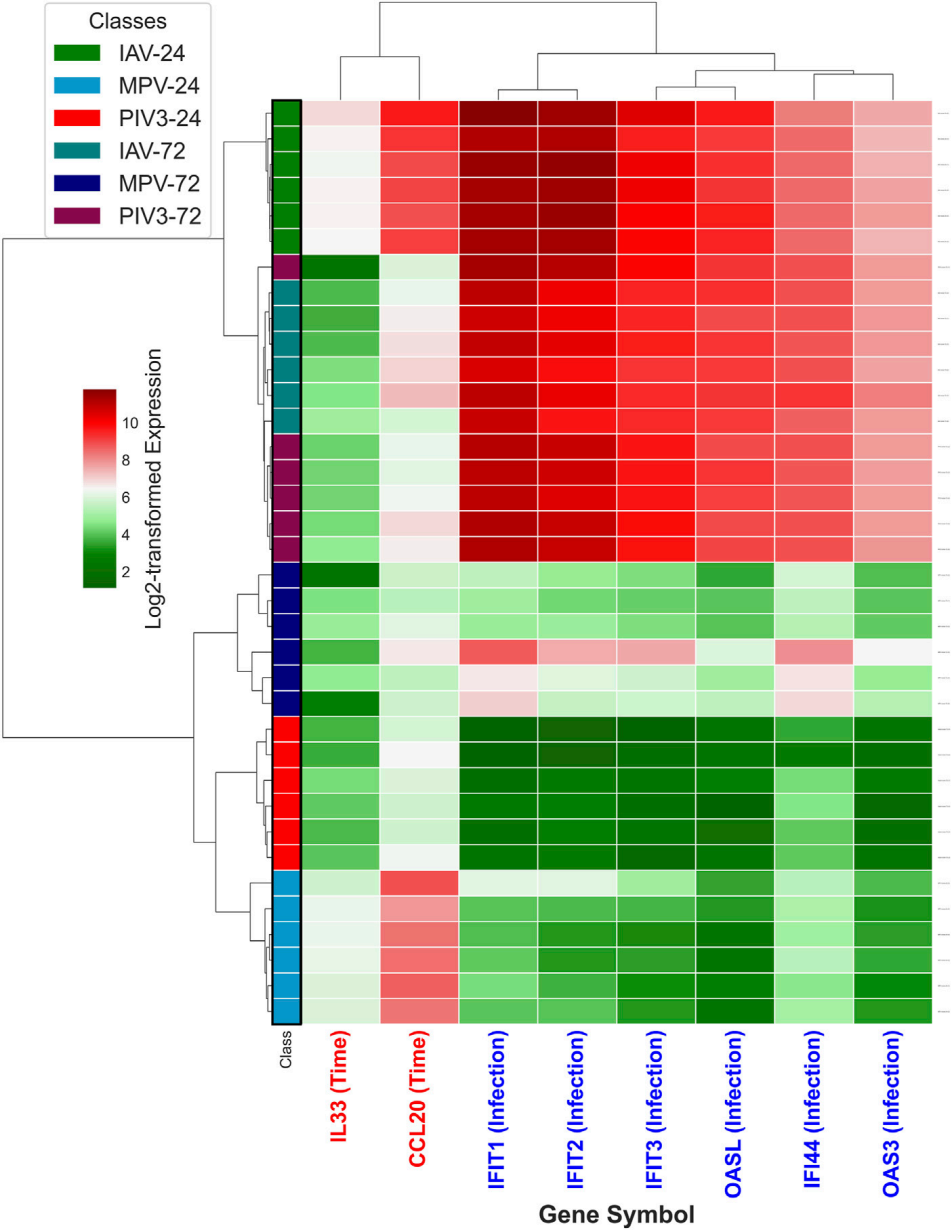
This figure illustrates the hierarchical clustering of all infected OTEs within the RNA-Seq dataset, using the eight genes identified through the MASIT algorithm applied exclusively to the NanoString dataset. The genes include six infection-dependent genes (IFIT1, IFIT2, IFIT3, OASL, OAS3 and IFI44) marked with blue in the annotation, and two time-dependent genes (IL33, CCL20) highlighted in red.

It turns out that the top three selected infection-dependent genes at time 24 are *IFIT1*, *IFIT2*, and *IFIT3*, and at time 72, they are *IFI44*, *OAS3*, and *OASL*. Additionally, MASIT was applied to identify time-dependent genes across the entire NanoString dataset, resulting in the selection of *IL33* and *CCL20* as the top time-dependent genes. Following this selection process, which was conducted exclusively on the NanoString data and without prior exposure to the RNA-Seq data (comprising 19,671 genes), Figure 2 illustrates the hierarchical clustering of all infected OTEs within the RNA-Seq dataset using these eight specifically selected genes.

### 3.2 Cross-modal predictive modeling with MASIT

In this section, we apply MASIT to NanoString data as described in Section 2.4. Our approach uses a 6-fold stratified cross-validation to train and validate models that simultaneously classify infections by multiple viruses: IAV, MPV, and PIV3. These models determine whether the OTEs are infected and, if so, identify the specific virus and ascertain the post-infection times of 24 and 72 h.

We refer to our models as MASIT-model, reflecting the integration of the MASIT process with various classification models. Each model begins by applying MASIT to the training set to identify the most statistically significant infection-dependent genes, as well as time-dependent genes. These genes are then employed as predictors (input features) for predictive models, including Logistic Regression in both one-vs-one (OVO) and one-vs-rest (OVR) configurations, and for SVM with OVO and OVR strategies using linear kernels. Additional models used include Support Vector Machines (SVM) with a linear kernel, Naive Bayes, Random Forest (RF), XGBoost, AdaBoost, Gradient Boosting Machine (GB), Extremely Randomized Trees (ER), and k-Nearest Neighbors (kNN). The efficacy of these models is evaluated on the validation set using only the genes selected during the training phase.

To ensure maximum generalizability and robustness of our models, we incorporated bootstrapping (Kohavi, 1995) into our cross-validation process. This involves randomly resampling the training dataset with replacement to create multiple bootstrap datasets. For each bootstrap sample, the MASIT process identifies key genes, and models are subsequently trained. This repetition helps stabilize the predictive power of the models by mitigating overfitting and providing a more reliable estimate of model performance.

**FIGURE 3:**
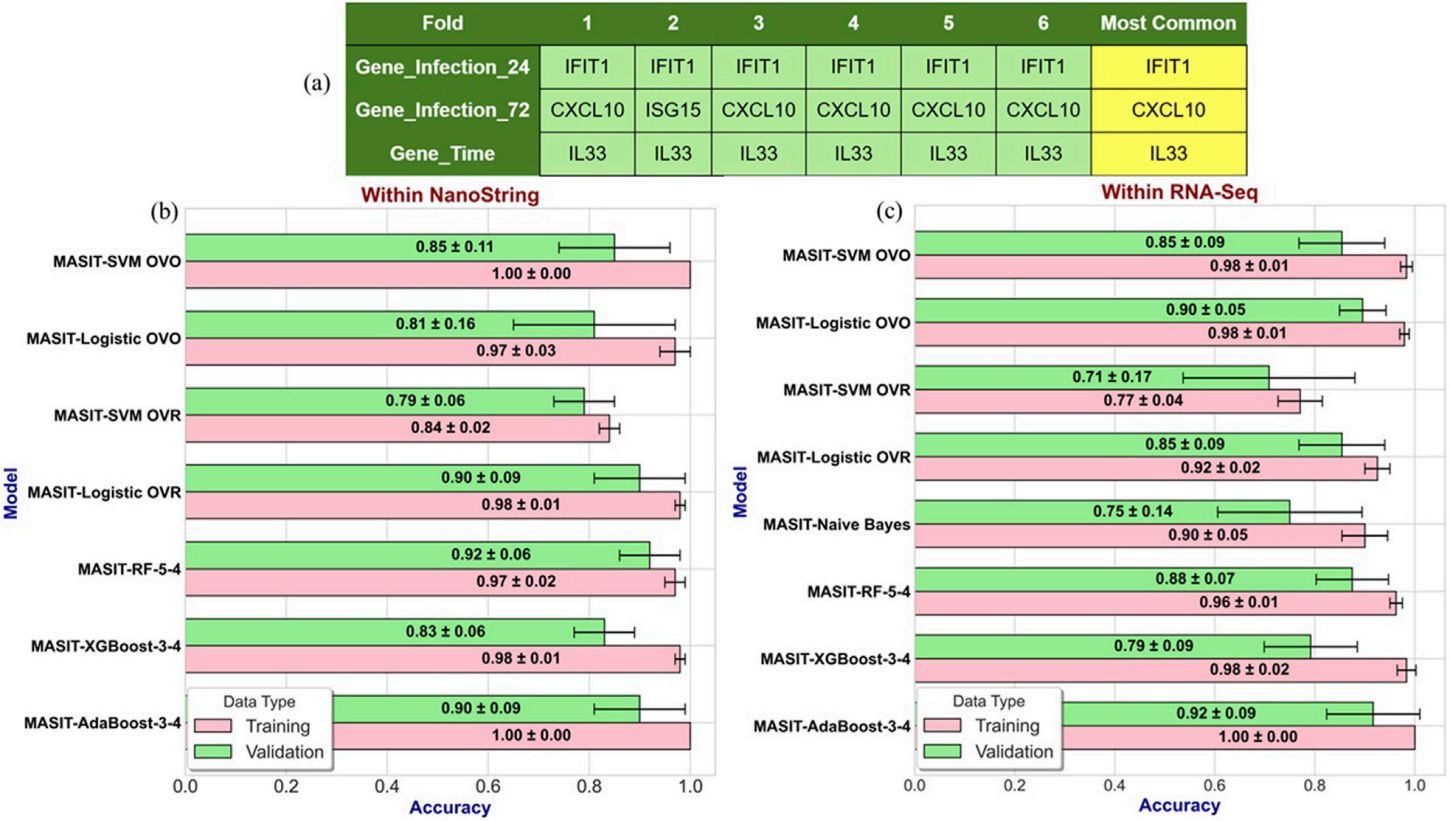
**(a)** Illustrates the infection- and time-dependent genes within Nanostring data for each fold within the 6-fold stratified cross-validation strategy, where we identified IFIT1 and CXCL10 as top infection-dependent genes at 24- and 72-h post-infection, respectively, and IL33 as the top time-dependent gene. **(b,c)** illustrate the performance accuracies of various models using MASIT-selected genes (IFIT1, CXCL10, and IL33) on both NanoString **(b)** and RNA-Seq **(c)** datasets. The models evaluated include SVM, LR, Random Forest with 5 trees and a maximum depth of 4 (MASIT-RF-5-4), XGBoost with 3 trees and a maximum depth of 4 (MASIT-XGBoost-3-4), and AdaBoost with 3 trees and a maximum depth of 4 (MASIT-AdaBoost-3-4).

Figure 3a illustrates the infection and time-dependent genes for each fold within the 6-fold stratified cross-validation strategy, where we identified *IFIT1* and *CXCL10* as top infection-dependent genes for 24- and 72-h post-infection, respectively, and *IL33* as the top time-dependent gene. In Figure 3b, the effectiveness of models such as SVM, LR, and three variants of tree-based algorithms using MASIT-selected genes as input features are demonstrated using the NanoString dataset.

Note that in our previous studies (Rezapour et al., 2024a; Rezapour et al., 2024b; Rezapour M. et al., 2024), we have comprehensively discussed the set of biomarkers for each virus. However, our goal here is to identify the top infection and time-dependent genes that can effectively identify the infection and time status even for unseen data. Table 2 shows the training and validation accuracies of the 6-fold cross-validation for various classifiers using the MASIT-selected genes within on NanoString data. The models evaluated include Random Forest (RF), XGBoost, AdaBoost (AB), Gradient Boosting (GB), and Extremely Randomized Trees (ET) with varying numbers of trees and maximum depths, as well as Support Vector Machines (SVM) with both one-vs-one (OVO) and one-vs-rest (OVR) configurations, Logistic Regression (LR) with OVO and OVR, Naive Bayes, and k-Nearest Neighbors (kNN). Each model’s performance is reported as the mean ± standard deviation for both training and validation sets.

**TABLE 2:** This table presents the training and validation accuracies obtained from 6-fold cross-validation of different classifiers using the genes selected by the MASIT algorithm within each fold of the NanoString dataset.

Model	MASIT- RF-2-2	MASIT- RF-3-2	MASIT- RF-4-2	MASIT- RF-5-2	MASIT- RF-6-2	MASIT- RF-7-2
Training	0.71 ± 0.09	0.73 ± 0.09	0.76 ± 0.05	0.87 ± 0.05	0.90 ± 0.03	0.91 ± 0.03
Validation	0.67 ± 0.16	0.73 ± 0.20	0.77 ± 0.17	0.77 ± 0.13	0.81 ± 0.16	0.81 ± 0.16
Model	MASIT- RF-2-3	MASIT- RF-3-3	MASIT- RF-4-3	MASIT- RF-5-3	MASIT- RF-6-3	MASIT- RF-7-3
Training	0.71 ± 0.09	0.73 ± 0.09	0.76 ± 0.05	0.87 ± 0.05	0.90 ± 0.03	0.91 ± 0.03
Validation	0.67 ± 0.16	0.73 ± 0.20	0.77 ± 0.17	0.77 ± 0.13	0.81 ± 0.16	0.81 ± 0.16
Model	MASIT- RF-2-4	MASIT- RF-3-4	MASIT- RF-4-4	MASIT- RF-5-4	MASIT- RF-6-4	MASIT- RF-7-4
Training	0.93 ± 0.03	0.95 ± 0.02	0.97 ± 0.02	0.97 ± 0.02	0.97 ± 0.02	0.97 ± 0.02
Validation	0.85 ± 0.13	0.81 ± 0.12	0.83 ± 0.06	**0.92** ± **0.06**	0.88 ± 0.10	0.90 ± 0.09
Model	MASIT- XGBoost-2-2	MASIT- XGBoost-3-2	MASIT- XGBoost-4-2	MASIT- XGBoost-5-2	MASIT- XGBoost-6-2	MASIT- XGBoost-7-2
Training	0.98 ± 0.01	0.98 ± 0.01	0.98 ± 0.01	0.98 ± 0.01	0.99 ± 0.01	1.00 ± 0.00
Validation	0.81 ± 0.06	0.83 ± 0.06	0.81 ± 0.06	0.81 ± 0.06	0.81 ± 0.06	0.81 ± 0.06
Model	MASIT- XGBoost-2-3	MASIT- XGBoost-3-3	MASIT- XGBoost-4-3	MASIT- XGBoost-5-3	MASIT- XGBoost-6-3	MASIT- XGBoost-7-3
Training	0.98 ± 0.01	0.98 ± 0.01	0.98 ± 0.01	0.98 ± 0.01	0.99 ± 0.01	1.00 ± 0.00
Validation	0.81 ± 0.06	0.83 ± 0.06	0.81 ± 0.06	0.81 ± 0.06	0.81 ± 0.06	0.81 ± 0.06
Model	MASIT- XGBoost-2-4	MASIT- XGBoost-3-4	MASIT- XGBoost-4-4	MASIT- XGBoost-5-4	MASIT- XGBoost-6-4	MASIT- XGBoost-7-4
Training	0.98 ± 0.01	0.98 ± 0.01	0.98 ± 0.01	0.98 ± 0.01	0.99 ± 0.01	1.00 ± 0.00
Validation	0.81 ± 0.06	0.83 ± 0.06	0.81 ± 0.06	0.81 ± 0.06	0.81 ± 0.06	0.81 ± 0.06
Model	MASIT- AB-2-2	MASIT- AB -3-2	MASIT- AB -4-2	MASIT- AB -5-2	MASIT- AB -6-2	MASIT- AB -7-2
Training	0.93 ± 0.03	0.98 ± 0.01	0.98 ± 0.01	0.98 ± 0.01	0.98 ± 0.01	0.98 ± 0.01
Validation	0.83 ± 0.09	0.88 ± 0.07	0.88 ± 0.07	0.85 ± 0.09	0.85 ± 0.09	0.90 ± 0.05
Model	MASIT- AB -2-3	MASIT- AB -3-3	MASIT- AB -4-3	MASIT- AB -5-3	MASIT- AB -6-3	MASIT- AB -7-3
Training	1.00 ± 0.00	1.00 ± 0.00	1.00 ± 0.00	1.00 ± 0.00	1.00 ± 0.00	1.00 ± 0.00
Validation	0.88 ± 0.07	0.90 ± 0.09	0.90 ± 0.09	0.90 ± 0.09	0.85 ± 0.11	0.92 ± 0.09
Model	MASIT- AB -2-4	MASIT- AB -3-4	MASIT- AB -4-4	MASIT- AB -5-4	MASIT- AB -6-4	MASIT- AB -7-4
Training	1.00 ± 0.00	1.00 ± 0.00	1.00 ± 0.00	1.00 ± 0.00	1.00 ± 0.00	1.00 ± 0.00
Validation	0.88 ± 0.07	0.90 ± 0.09	0.90 ± 0.09	0.90 ± 0.09	0.85 ± 0.11	0.92 ± 0.09
Model	MASIT- GB-2-2	MASIT- GB -3-2	MASIT- GB -4-2	MASIT- GB -5-2	MASIT- GB -6-2	MASIT- GB -7-2
Training	1.00 ± 0.00	1.00 ± 0.00	1.00 ± 0.00	1.00 ± 0.00	1.00 ± 0.00	1.00 ± 0.00
Validation	0.83 ± 0.06	0.81 ± 0.06	0.81 ± 0.06	0.81 ± 0.06	0.83 ± 0.06	0.83 ± 0.06
Model	MASIT- GB -2-3	MASIT- GB -3-3	MASIT- GB 4-3	MASIT- GB -5-3	MASIT- GB -6-3	MASIT- GB -7-3
Training	1.00 ± 0.00	1.00 ± 0.00	1.00 ± 0.00	1.00 ± 0.00	1.00 ± 0.00	1.00 ± 0.00
Validation	0.83 ± 0.06	0.81 ± 0.06	0.81 ± 0.06	0.81 ± 0.06	0.83 ± 0.06	0.83 ± 0.06
Model	MASIT- GB -2-4	MASIT- GB -3-4	MASIT- GB -4-4	MASIT- GB -5-4	MASIT- GB -6-4	MASIT- GB -7-4
Training	1.00 ± 0.00	1.00 ± 0.00	1.00 ± 0.00	1.00 ± 0.00	1.00 ± 0.00	1.00 ± 0.00
Validation	0.81 ± 0.10	0.81 ± 0.10	0.81 ± 0.10	0.83 ± 0.06	0.81 ± 0.10	0.81 ± 0.10
Model	MASIT- ET-5-2	MASIT- ET-5-3	MASIT- ET-5-4	MASIT- ET-6-2	MASIT- ET-6-3	MASIT- ET-6-4
Training	0.75 ± 0.06	0.75 ± 0.06	0.85 ± 0.02	0.76 ± 0.05	0.76 ± 0.05	0.85 ± 0.01
Validation	0.73 ± 0.09	0.73 ± 0.09	0.77 ± 0.09	0.71 ± 0.09	0.71 ± 0.09	0.75 ± 0.07
Model	MASIT- SVM OVO	MASIT- SVM OVR	MASIT- LR OVO	MASIT- LR OVR	MASIT- Naive Bayes	MASIT-kNN-3
Training	1.00 ± 0.00	0.84 ± 0.02	0.97 ± 0.03	0.98 ± 0.01	0.97 ± 0.02	0.89 ± 0.02
Validation	0.85 ± 0.11	0.79 ± 0.06	0.81 ± 0.16	0.90 ± 0.09	0.88 ± 0.10	0.81 ± 0.10

The classifiers evaluated include Random Forest (RF), XGBoost, AdaBoost (AB), Gradient Boosting (GB), and Extremely Randomized Trees (ET) with varying numbers of trees and maximum depths, as well as Support Vector Machines (SVM) in both one-vs-one (OVO) and one-vs-rest (OVR) configurations, Logistic Regression (LR) with OVO and OVR, Naive Bayes, and k-Nearest Neighbors (kNN). Each model’s performance is reported as the mean accuracy ± standard deviation for both the training and validation sets. The notation MASIT-Model-m-n indicates that the MASIT algorithm was first applied to the training set to identify the top infection and time-dependent genes, and then these genes were used as predictors for the model, with “m” representing the number of trees and “n” representing the maximum depth of the trees.

Bold values indicate better performance.

Using the most common MASIT-selected genes within all folds, *IFIT1* (infection dependent), *CXCL10* (infection dependent), and *IL33* (time dependent), we applied SVM (ovo and ovr), LR (ovo and ovr), Random Forest with 5 trees and a maximum depth of 4 (MASIT-RF-5-4), XGBoost with 3 trees and a maximum depth of 4 (MASIT-XGBoost-3-4), and AdaBoost with 3 trees and a maximum depth of 4 (MASIT-AdaBoost-3-4) on the held-out RNA-Seq samples using the same 6-fold cross-validation to compare the results of classification within RNA-Seq and NanoString using the MASIT-selected genes from NanoString. To further evaluate the model’s robustness, we applied a similar bootstrapping strategy to the RNA-Seq dataset. Figure 3c shows the performance of the models using the MASIT-selected genes from NanoString data on the held-out RNA-Seq dataset, which illustrates the cross-platform validation of predictive capabilities using these key genes.

**FIGURE 4:**
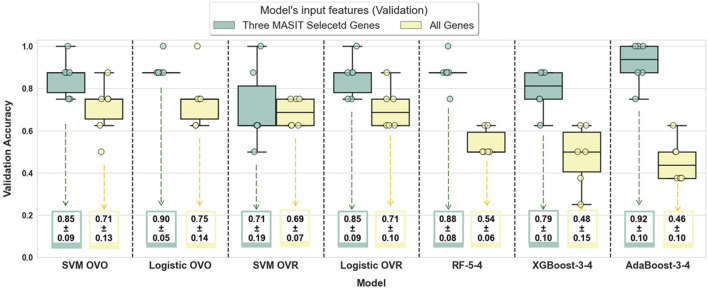
This figure compares the validation accuracies of various models trained using MASIT-selected genes (IFIT1, CXCL10, and IL33) and the entire gene set (19,671 genes) within RNA-Seq data. The models evaluated include SVM with OVO and OVR configurations, Logistic Regression with OVO and OVR configurations, Random Forest with 5 trees and a maximum depth of 4 (RF-5-4), XGBoost with 3 trees and a maximum depth of 4 (XGBoost-3-4), and AdaBoost with 3 trees and a maximum depth of 4 (AdaBoost-3-4).

To illustrate the efficiency of MASIT-selected genes in classification performance, we also used the entire gene set within RNA-Seq (comprising 19,671 genes) to train and validate the performance of these models. Figure 4 illustrates the validation accuracies for both strategies: using MASIT-selected genes (only three genes, which helps avoid overfitting) and the entire gene set for all 6 folds.

**FIGURE 5:**
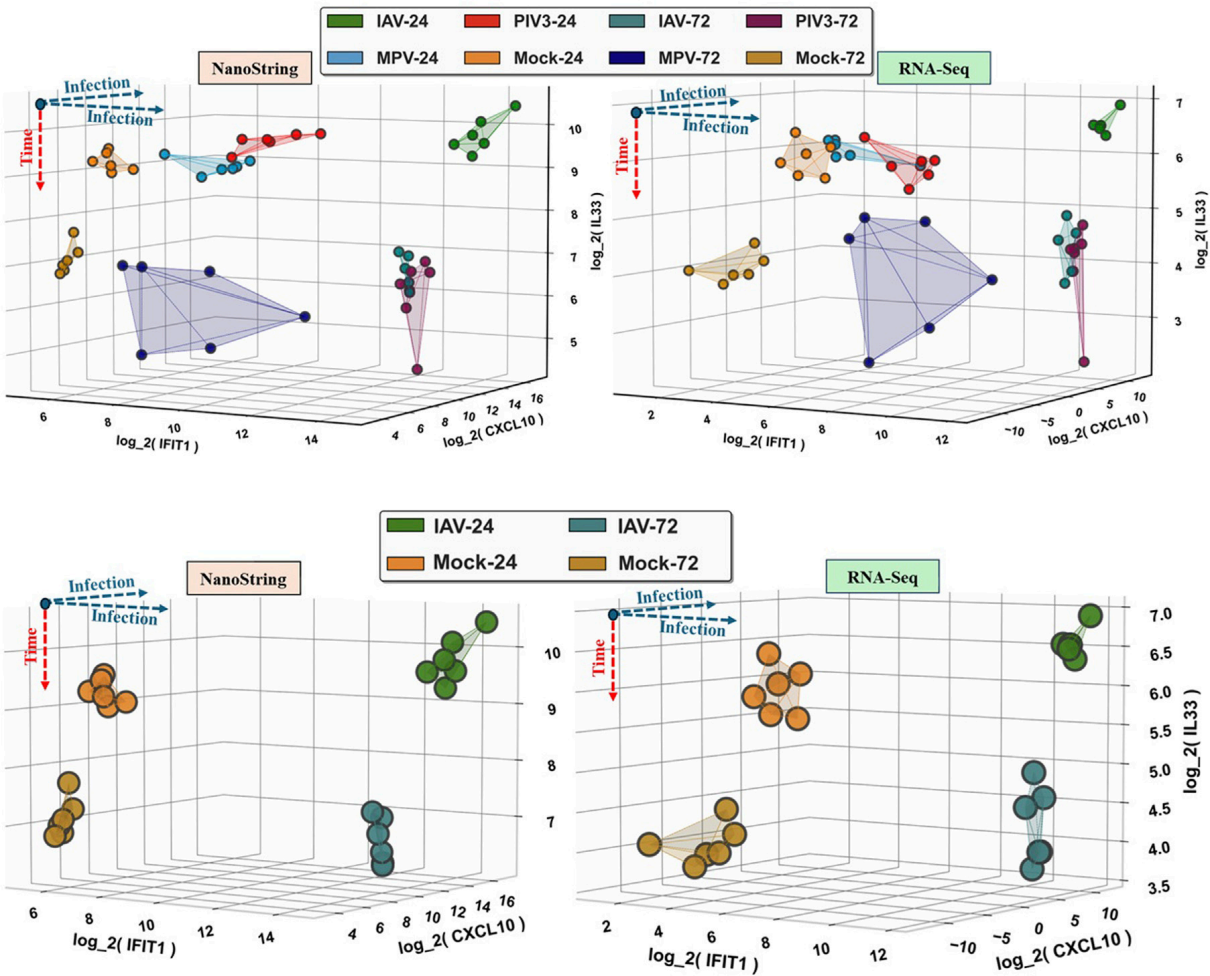
This figure provides a 3D visualization comparing the log2-transformed expression of IFIT1, CXCL10, and IL33 between NanoString and RNA-Seq datasets. The left panels represent the NanoString data, while the right panels represent the RNA-Seq data. The top panels show all samples, and the bottom panels focus specifically on IAV-infected samples and Mock samples as examples. Each axis corresponds to the log2-transformed expression levels of one of the three genes.

To compare the expression of these three genes, *IFIT1*, *CXCL10*, and *IL33* between NanoString and RNA-Seq, Figure 5 provides a 3D visualization of all samples using *IFIT1*, *CXCL10*, and *IL33* as the coordinate axes. The left panels are for NanoString and the right panels are for RNA-Seq, where the top panels visualize all samples, and the bottom panels specifically show only IAV-infected samples and Mock samples as examples.

**FIGURE 6:**
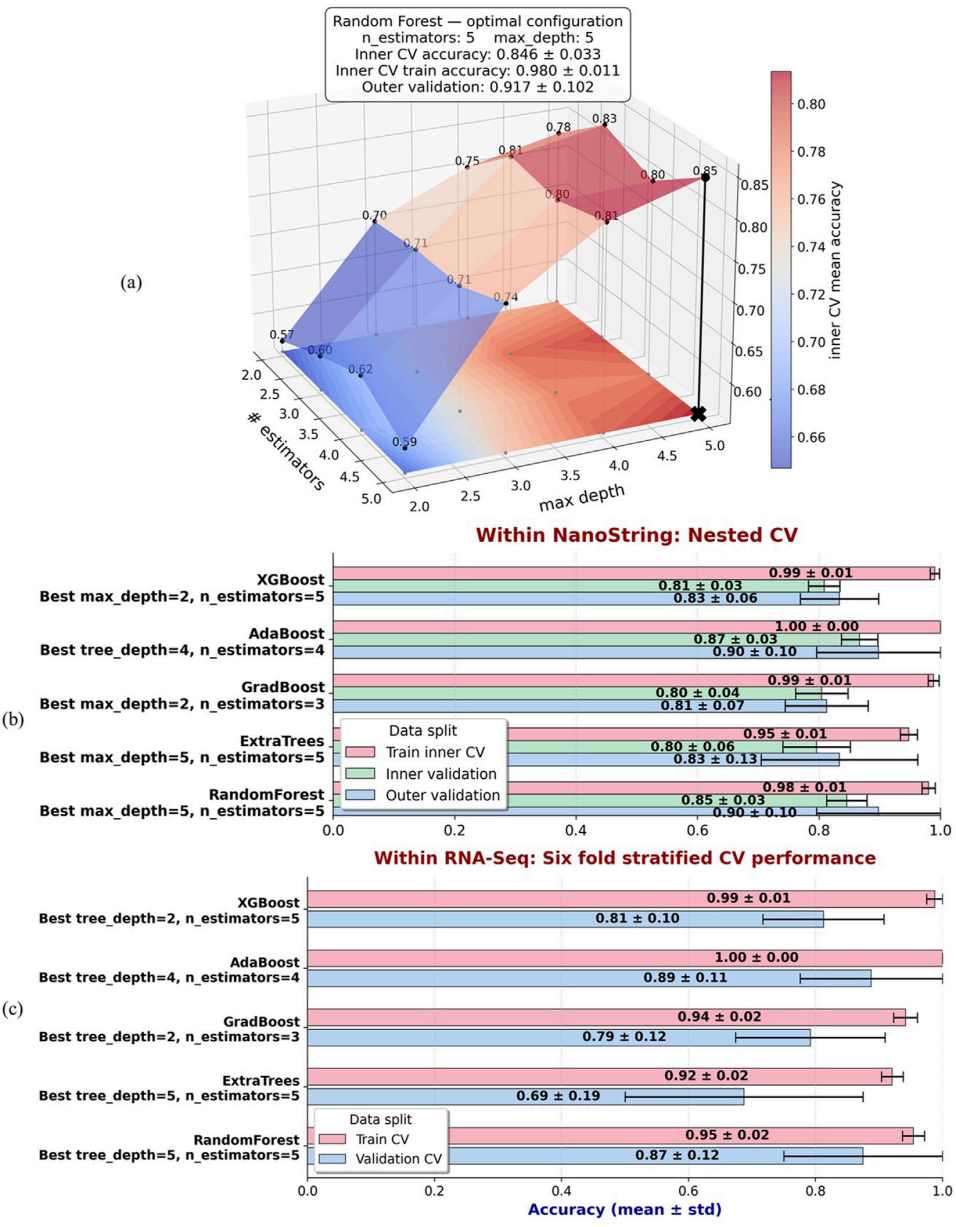
**(a)** Nested cross validation surface of Random Forest performance within the NanoString dataset across a grid of the number of estimators and maximum depth. The color surface represents the mean inner validation accuracy, averaged across outer folds. The black cross denotes the optimal configuration, identified as six estimators with a maximum depth of five, based on the highest inner validation mean accuracy. Reported metrics include mean ± standard deviation for training accuracy (averaged across inner training folds), inner validation accuracy (averaged across inner validation folds), and outer validation accuracy (averaged across outer folds at the optimal configuration). **(b)** Comparative results of nested cross validation for tree-based ensemble models, including Random Forest, Extra Trees, Gradient Boosting, AdaBoost, and XGBoost, within the NanoString dataset. For each model, the optimal hyperparameters were selected by the inner grid search. Bars show mean accuracy ± standard deviation for training, inner validation, and outer validation performance at these optimal configurations. **(c)** Performance of the same models on RNA-Seq data using MASIT-selected genes from the NanoString dataset. For this analysis, hyperparameters were fixed to the optimal values previously identified within NanoString (panel b), and models were evaluated using 6-fold stratified cross validation.

To assess how the performance of tree-based ensemble models changes with model capacity, we performed nested cross-validation over a grid of the number of estimators and maximum tree depth. Figure 6a shows the resulting inner-CV mean-accuracy surface for the Random Forest model. The black cross marks the optimal configuration, which was identified as six estimators (trees) with a maximum depth of five, based on the highest inner validation mean accuracy. In this framework, “training accuracy” refers to the average score obtained on the inner training folds during the grid search, “inner validation accuracy” refers to the average score on the held out inner folds used for tuning, and “outer validation accuracy” is computed by refitting the model with the inner optimal parameters on each outer training set and scoring on the corresponding outer test set, which provides. For each metric, the reported mean is the arithmetic average across folds, and the standard deviation quantifies variability across folds.

We then applied the same nested cross validation procedure to other tree-based ensemble models, including Extra Trees, Gradient Boosting, AdaBoost, and XGBoost. For each model, the inner grid search identified the hyperparameter configuration that maximized mean inner validation accuracy, after which performance was summarized across training folds, inner validation folds, and outer validation folds. Figure 6b presents a comparative overview of these models, which shows mean accuracy and standard deviation for training, inner validation, and outer validation under their respective optimal configurations.

Finally, to assess how the NanoString-derived optimal configurations generalize to RNA-Seq data, we applied the same models using the hyperparameters selected from the NanoString nested cross validation. In this case, we did not repeat nested cross validation for the RNA-Seq dataset. Instead, we directly evaluated each model using 6-fold stratified cross validation on the RNA-Seq data, restricted to the same MASIT-selected genes and the optimal configuration for the model learned from NanoString. Figure 6c shows these results, which provide a direct comparison of model performance when transferring NanoString-optimized hyperparameters and gene features to RNA-Seq data.

Finally, to directly compare MASIT with existing feature selection approaches, we replaced the MAS component of MASIT with Fisher score, mRMR, embedded Lasso, or Boruta, while keeping the remainder of the framework identical. To minimize overfitting in this small-sample setting, each method was constrained to select only three genes per fold within the six-fold stratified cross-validation on NanoString data, and Random Forest classifiers were tuned via nested cross-validation using the number of trees and maximum depth.

**FIGURE 7:**
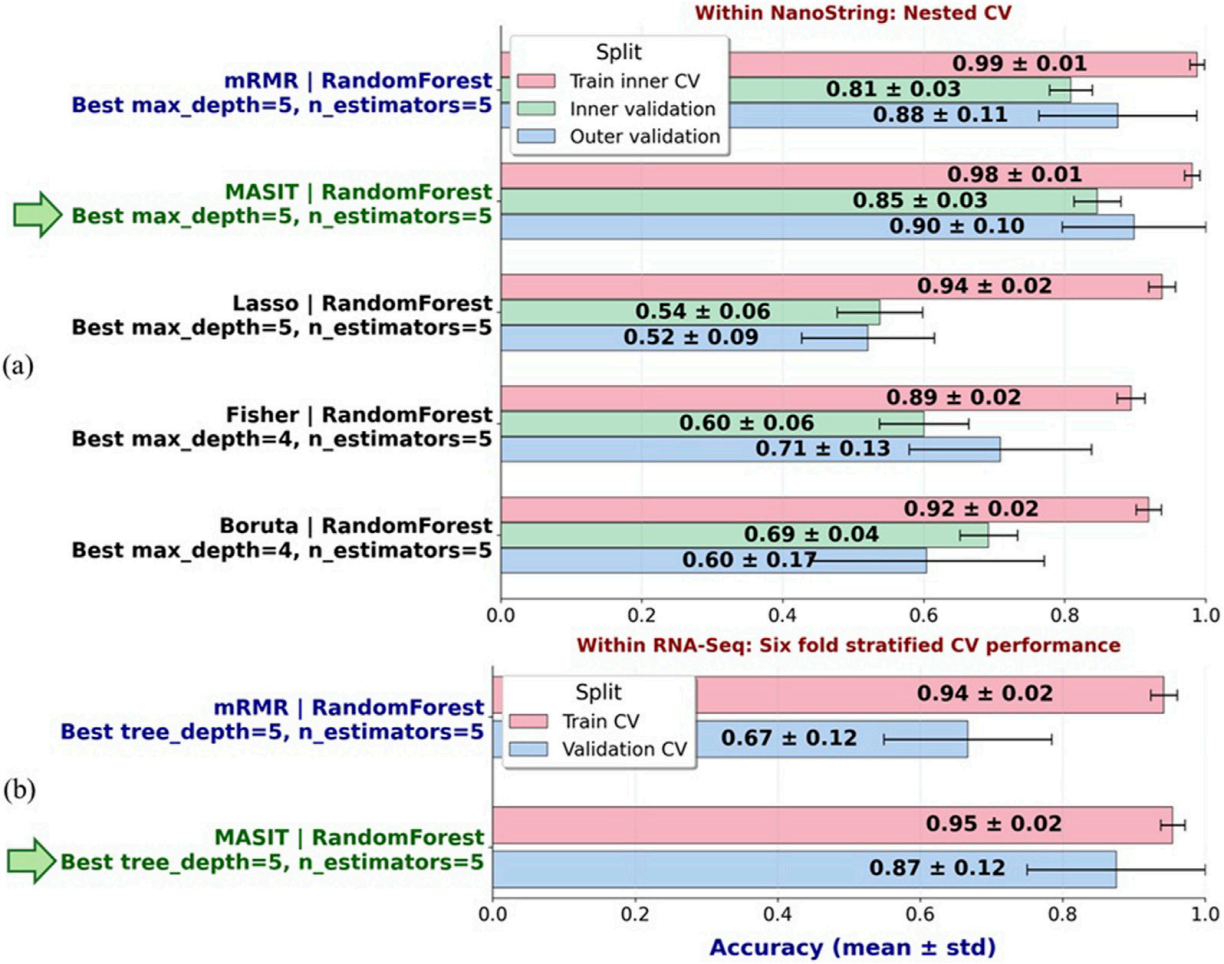
**(a)** Nested cross-validation results within NanoString data using Random Forest classifiers with tuned hyperparameters (tree depth and number of estimators). MASIT and mRMR achieved the highest outer validation accuracies (0.90 ± 0.10 and 0.88 ± 0.11, respectively), while Fisher, Lasso, and Boruta yielded lower accuracies ranging from 0.52 to 0.71. Bars show mean ± standard deviation across folds for training, inner validation, and outer validation accuracies. **(b)** Cross-platform evaluation of MASIT and mRMR on the held-out RNA-Seq dataset. Using the optimal Random Forest configuration identified from NanoString (tree depth = 5, estimators = 5), genes selected by each method were directly applied to RNA-Seq. MASIT achieved a substantially higher validation accuracy (0.87 ± 0.12) compared to mRMR (0.67 ± 0.12), while both methods maintained comparable training performance.

Figure 7a summarizes the optimal configurations and performance for each method. Within NanoString data, MASIT and mRMR achieved the highest outer validation accuracies (0.90 ± 0.10 and 0.88 ± 0.11, respectively), whereas Lasso, Fisher, and Boruta produced substantially lower accuracies (ranging from 0.52 to 0.71). To further assess cross-platform robustness, we selected the two best-performing feature selection strategies from the NanoString experiments, MASIT and mRMR, and applied them directly to the held-out RNA-Seq dataset. Using the same Random Forest classifier with the optimal hyperparameters identified in the NanoString nested cross-validation (tree depth = 5, number of estimators = 5), we evaluated whether the selected genes could generalize to the broader transcriptome. As shown in Figure 7b, MASIT achieved a considerably higher validation accuracy (0.87 ± 0.12) compared to mRMR (0.67 ± 0.12), while maintaining similar training performance. These findings highlight MASIT’s ability not only to match state-of-the-art methods within NanoString but also to transfer effectively across platforms.

## 4 Discussion

### 4.1 Cross-modal capability of MASIT

The robustness of the MASIT in selecting statistically significant genes is exemplified by its success in identifying key markers for viral infection states and their progression over time. The MASIT selected genes, *IFIT1*, *IFIT2*, *IFIT3*, *OASL*, *IFI44*, *OAS3*, *IL33*, and *CCL20* from NanoString data, not only effectively categorize RNA-Seq samples by viral type and infection stage as shown in Figure 2 but also offer insights into the host/OTE’s molecular response to viral infections.

The IFIT family, comprising *IFIT1*, and *IFIT3*, plays a key role in the antiviral defense, particularly against RNA viruses such as IAV, MPV, and PIV3. These proteins, by binding to viral RNA with a triphosphate group (PPP-RNA), prevent viral replication and translation, and showcase a specific defense against viruses that utilize PPP-RNA structures, such as IAV (Pichlmair et al., 2011). Moreover, the interaction of IFIT proteins with the 5′ cap of viral RNA blocks the translation of viral proteins is important for regulating the host’s immune response via the JAK-STAT signaling pathway (Zhou et al., 2013). This mechanism not only underscores the adaptability of IFIT proteins in controlling viral infections but also enhances our understanding of their broader role in immune regulation. Fensterl et al. (Fensterl and Sen, 2015) contributed additional insights into the non-enzymatic antiviral mechanisms of IFITs, highlighting how these proteins inhibit virus replication by binding and sequestering RNA molecules from viruses and modulating protein interactions.

*OASL* and *OAS3* are significant for their roles in the immune response to viral infections. *OAS3*’s activation by double-stranded RNA (dsRNA), typical of viral replication intermediates, and its subsequent induction of RNase L lead to the degradation of viral RNA, effectively stopping virus replication (Choi et al., 2015). This role is important in combating viruses like IAV. *OASL*, on the other hand, enhances the RIG-I signaling pathway that is important during the early stages of viral infections, and its ability to modulate RIG-I sensitivity significantly impacts the immune response to viral infection (Melchjorsen et al., 2009).

*IFI44*, highlighted by its upregulation in conditions such as respiratory syncytial virus (RSV) infections, plays a role in the formation of microtubular structures and pathways activated by IFN-α stimulation. This positions *IFI44* as a potential therapeutic target and a biomarker for RSV infections, providing insights into its dual role in enhancing and suppressing immune activity (Li et al., 2021; DeDiego et al., 2019).

*IL33* and *CCL20*, selected as time-dependent genes, reveal the MASIT’s ability to recognize genes whose expression patterns are indicative of specific physiological or immunological events. *IL33*, for instance, is important in type-2 immune activation and plays a significant role in allergic lung inflammation, which aligns with its temporal expression patterns (Hardman et al., 2013). *CCL20*’s upregulation in response to inflammatory stimuli further underscores the model’s utility in capturing key temporal dynamics related to immune responses (Schutyser et al., 2003).

The clustering in Figure 2 validates the MASIT’s effectiveness in identifying relevant biomarkers that maintain consistency across NanoString and RNA-Seq platforms. This versatility in gene expression profiling technologies demonstrates the model’s utility in providing comprehensive insights into the OTE (host)’s molecular response to infections. The ability to identify and validate these key biomarkers across platforms not only enhances our understanding of viral pathogenesis but also strengthens the MASIT framework’s application in future infectious disease research and therapeutic development.

### 4.2 Cross-modal predictive modeling with MASIT

Table 2 illustrates the performance of several machine learning classifiers on NanoString data, using only three MASIT-selected genes from each fold’s training set. To better understand MASIT’s efficiency, we selected a range of models, including both linear and some tree-based ensemble models that demonstrated strong performance during validation. These models include MASIT-SVM (OVO and OVR) with a linear kernel, MASIT-LR (OVO and OVR), Random Forest with five trees and a maximum depth of four (MASIT-RF-5-4), XGBoost with three trees and a maximum depth of four (MASIT-XGBoost-3-4), and AdaBoost with three trees and a maximum depth of four (MASIT-AdaBoost-3-4). We compared the results of 8-class classification on the RNA-Seq and NanoString data, which are visualized in Figure 3.

Using only three MASIT-selected genes as predictors in classifiers, particularly for linear models such as SVM and Logistic Regression, as well as tree-based ensemble models with few estimators (trees), significantly reduced the risk of overfitting. This limitation in the models’ complexity, combined with a small number of predictors, inherently lowers the variance and prevents the models from learning noise and idiosyncrasies in the data. This focused approach not only enhances model generalizability but also maintains robustness in prediction accuracy. The alignment of training and validation performances for models utilizing MASIT-selected genes, as illustrated in Figure 3, validates the efficacy of MASIT in identifying the most informative and generalizable features from complex genomic data.

Figure 4 showcases the validation accuracies for various classifiers using either three MASIT-selected genes or all genes within the RNA-Seq dataset, which highlights a clear contrast in performance between more focused and extensive feature sets. The box plots illustrate a trend where models using just three MASIT selected genes generally achieve higher median validation accuracies compared to those using all genes. This difference is particularly notable in complex models such as Random Forest, XGBoost, and AdaBoost, where the performance with all genes markedly decreases, which signals potential overfitting when the feature space is too large. In contrast, the simpler, linear models (SVM and Logistic Regression) demonstrate less variation in performance between the two feature sets, though they still benefit from the reduced complexity afforded by using only the selected genes.

Additionally, the use of only three MASIT-selected genes leads to more stable and consistent validation performances across all models. This suggests that the MASIT selection process effectively captures the most predictive features, thereby enhancing the models’ ability to generalize without being misled by the noise and redundancy often present in larger datasets.

Moreover, the significant disparity in performance between the full gene set and the MASIT-selected gene set, highlighted in Figure 4, underscores the tendency for complex models to overfit when equipped with unrestricted gene sets. Notably, when all genes were used in RNA-Seq, overfitting occurred across all models because we observed 100% accuracies in training, while the validation performances did not match those of training, indicating a significant generalization gap. This demonstrates the risk associated with using a large number of predictors and the benefits of a more targeted approach. Therefore, MASIT’s method of selecting a concise, impactful set of genes is a key strategy in genomic studies that facilitate the development of robust, accurate, and generalizable models, and thus establishing itself as an invaluable tool in the field of genomics.

Figure 5 demonstrates that both NanoString and RNA-Seq platforms provide consistent and comparable expression data for *IFIT1*, *CXCL10*, and *IL33*. The 3D visualization emphasizes the ability of these genes to serve as biomarkers for infection status and progression. This comparative analysis underscores the robustness of using these genes for studying infection dynamics and highlights the effectiveness of both NanoString and RNA-Seq technologies in capturing gene expression profiles.

These findings, supported by the comparative analyses shown in Figures 3–5, suggest that employing MASIT for feature selection should be considered a best practice in genomic model training, especially when addressing high-dimensional data where overfitting poses a significant risk. This approach does not merely simplify the training process but also ensures the relevance and reliability of the models in real-world applications.

The comparative evaluation of tree-based ensemble classifiers in Figure 6 highlights that Random Forest, XGBoost, and AdaBoost consistently achieved strong validation performance when applied to MASIT-selected features. This indicates that MASIT enhances predictive accuracy across different ensemble modeling frameworks, which demonstrates that its utility is not confined to a single algorithm.

Figure 7 provides a structured comparison between MASIT and widely used feature selection approaches evaluated under identical modeling conditions. Within NanoString using nested cross validation, MASIT and mRMR achieved the highest outer validation accuracies, which were 0.90 ± 0.10 and 0.88 ± 0.11, respectively, whereas Fisher score, embedded Lasso, and Boruta produced substantially lower accuracies ranging from 0.52 to 0.71. The gap between training and outer validation for Fisher, Lasso, and Boruta indicates limited generalization.

A key distinction emerges in the cross platform experiment. When we transferred the feature subsets and the Random Forest configuration selected within NanoString to the held out RNA Seq dataset, MASIT maintained high validation accuracy of 0.87 ± 0.12, whereas mRMR dropped to 0.67 ± 0.12 despite similar training performance. This divergence suggests that MASIT’s selection strategy, which integrates effect size with multiple testing control and then enforces agreement across infection conditions and time points, yields features that are more stable to platform specific measurement differences.

These results support two practical advantages. First, MASIT matches or exceeds state of the art methods within NanoString while selecting a very small and interpretable set of genes. Second, MASIT generalizes across platforms, which directly addresses a common source of failure for many feature selection pipelines that exhibit platform specific bias. Together, these findings clarify MASIT’s intended contribution: it is a statistically driven pre filter that emphasizes reproducible effect under stringent error control, which improves external validity while keeping model capacity low.

### 4.3 Limitations of the study

This study has several limitations: First, the small number of biological replicates limits statistical power and may constrain the generalizability of the findings, a challenge common in multi-omics studies. Second, although MASIT reduces overfitting by prioritizing informative features, some residual risk of overfitting cannot be fully eliminated, particularly when applying complex classifiers. Third, while MASIT successfully transferred between NanoString and RNA-Seq platforms, broader cross-platform applications (e.g., to proteomic or metabolomic datasets) will require further validation and potential scaling adjustments. Finally, although the RNA-Seq dataset was held out for testing, its limited size may restrict the strength of claims regarding independence and reproducibility. These considerations highlight areas for future work to further validate and extend the MASIT framework.

## 5 Conclusion

In our study, we tackled the challenge of developing robust predictive models from multi-omics data, contending with the dual constraints of small sample sizes and expansive feature spaces. We introduced the novel Magnitude-Altitude Score Analysis for Tracking Infection and Time-Dependent Genes (MASIT) methodology to enhance the accuracy and generalization capabilities of our models. MASIT adeptly sifts through gene, transcript, or protein features, pinpointing those important for understanding the dynamic responses in the OTE model. Using MASIT, we explored gene expression changes in OTEs following exposure to Influenza A virus (IAV), Human metapneumovirus (MPV), and Parainfluenza virus type 3 (PIV3) at 24- and 72-h post-infection. This approach leveraged the complementary strengths of RNA-Seq and NanoString technologies: RNA-Seq provided a broad transcriptomic overview of 19,671 genes, while NanoString offered precise quantification of 773 targeted genes, enabling a focused and comprehensive analysis.

Our methodology incorporated a K-Fold Stratified Cross-validation within MASIT to systematically select the most informative genes, each marking distinct infection types and stages, and others serving as temporal markers between time points. This selective feature strategy significantly mitigated the risk of overfitting, a prevalent issue in models trained on large datasets with limited samples. The predictive models, enhanced by MASIT, employed a concise set of genes chosen through this rigorous process. These models exhibited superior performance and generalizability compared to those utilizing the full gene set, particularly in complex models like Random Forest, XGBoost, and AdaBoost. Key markers identified by MASIT, including *IFIT1*, *IFIT2*, *IFIT3*, *OASL*, *IFI44*, and *OAS3*, were instrumental in effectively categorizing both NanoString and RNA-Seq samples by viral type and infection stage. MASIT’s robust cross-modal capability extends to predictive modeling. By strategically selecting a small number of highly informative genes, MASIT reduces the common risk of overfitting in genomic studies with extensive datasets. This focused approach not only enhances the models’ generalizability but also maintains prediction accuracy across different data modalities. The alignment of training and validation performances further underscores MASIT’s efficiency in feature selection, and affirms its efficacy in maintaining robust predictive accuracy across different platforms.

A comparative analysis between MASIT selected genes and the full gene set underscores a marked contrast in performance, especially in complex models such as Random Forest, XGBoost, and AdaBoost. Using a concise set of MASIT selected genes leads to higher validation accuracies and more consistent performance across models, which highlights the effectiveness of MASIT in capturing the most predictive features essential for robust modeling. This strategy not only simplifies the training process but also ensures the relevance and reliability of the models, and establishes MASIT as an invaluable tool in genomics. The ability of MASIT to consistently identify and validate key biomarkers across NanoString and RNA-Seq platforms enhances our understanding of viral pathogenesis and strengthens the framework’s application in infectious disease research and therapeutic development. This underscores the important role of MASIT in advancing genomic research and its potential to significantly influence the development of diagnostic and therapeutic strategies in viral infections.

Benchmarking against widely used feature selection approaches, including Fisher score (filtering), mRMR (wrapper), Lasso (embedded), and Boruta (hybrid), further confirmed MASIT’s advantages: it not only exceeded their performance within NanoString data but also uniquely maintained high accuracy and reproducibility on held-out RNA-Seq data. These results underscore MASIT’s superior robustness, reproducibility, and cross-platform generalizability.

## Data Availability

All data supporting the findings of this study are available from the corresponding author upon reasonable request.
